# Effects of Halliwick-based aquatic exercise on social and motor skills of children with autism spectrum disorder: a pilot study

**DOI:** 10.3389/fpsyg.2026.1832876

**Published:** 2026-05-19

**Authors:** Yu Liu, Junyang Feng, Yantao Zhu, Chengyang Li, Yong Zhou, Xuebing Wang

**Affiliations:** School of Physical Education, Guangxi University, Nanning, China

**Keywords:** aquatic exercise, ASD, balance, motor skill, social interaction

## Abstract

Limited evidence is available regarding the effects and duration of Halliwick-based aquatic exercise intervention in children with autism spectrum disorder (ASD). This pilot study aimed to investigate the feasibility, initial effects and short-term persistence of Halliwick-based aquatic exercise on social and motor skills in children with ASD. Twelve children with ASD were randomly assigned into either intervention group or control group. Assessments were conducted at baseline (T0), after 6 weeks of intervention (T1), at the end of intervention (T2), and 4 weeks after cessation of the intervention (T3). Outcome measures included balance performance (standing on one leg with eyes open and closed, intensive Romberg tests), the Autism Treatment Evaluation Checklist (ATEC), Children’s Autism Rating Scale (CARS), Children’s Sensory Integration Rating Scale (CSIS), and the Humphries Assessment of Aquatic Readiness (HAAR). In the intervention group, ATEC health/behavior and total score were significantly reduced at T1 compared to T0, and speech/language/communication scores were significantly lower than those of the control group. CARS scores showed significant reductions at T1, T2, and T3 compared to T0. In contrast, CSIS scores, balance performance and HAAR scores demonstrated significant increases from T1 to T3 compared to both baseline and the control group. This pilot study suggested that a 12-week Halliwick-based aquatic exercise program may be associated with improvements in social and motor skills in children with ASD, with some benefits maintained for at least 4 weeks. Large-scale studies are needed to confirm the preliminary findings.

## 1 Introduction

Autism Spectrum Disorder (ASD) is a neurodevelopmental disorder characterized by deficits in social interaction, rigid behaviors, and communication difficulties (Ansari et al., 2021a; Munn et al., 2021). The overall prevalence of ASD among 8-year-old children is 27.6 per 1,000 (or 1 in 36) (Maenner et al., 2023) and autism has gradually emerged as a primary cause of mental disability in children, with its incidence steadily increasing (Talantseva et al., 2023). Recent neurobiological perspectives emphasize that these core deficits are often intertwined with significant motor impairments, affecting approximately 80% of children with ASD (Bhat, 2021). These impairments, ranging from postural instability to poor neuromuscular coordination, not only limit physical activity but also exacerbate social isolation and sedentary-related health risks (Dong et al., 2024). Crucially, motor proficiency may serve as a developmental scaffold for social-communicative growth (Bäckström et al., 2021), suggesting that motor-based interventions could yield cascading benefits across multiple developmental domains.

Several studies have investigated the effects of different types of exercise and health promotion interventions on the social skills of children with autism (Ji et al., 2022; Hynes and Block, 2023; Howells et al., 2020; Sarabzadeh et al., 2019). However, most of these exercises are land-based, and although they can improve gross motor function in children with ASD, they are hindered by environmental and psychological barriers such as discomfort, fatigue, and concerns about injury risk (Bremer et al., 2016; Phung and Goldberg, 2021; Jagadeesan et al., 2022).

Aquatic exercise offers a safe, low-impact, and sensorially unique alternative to land-based exercises for children with autism, with Halliwick method being a notable example (Vodakova et al., 2022; Ansari et al., 2021b). The Halliwick method is designed to facilitate basic independent swimming skills while enhancing motor skills and balance. Existing research on underwater movement based on Halliwick method mainly focus on children with conditions such as cerebral palsy, Parkinson’s disease, and stroke, although there have been investigations into underwater rehabilitation for autistic children (Rohn et al., 2021; Ansari et al., 2021b). Furthermore, studies have indicated that Halliwick method combining water environment with exercise training may exert therapeutic effects on autistic children (Vodakova et al., 2022; Rohn et al., 2021). However, a recent systematic review suggested that the efficacy of Halliwick-based aquatic interventions remains inconsistent, with some studies reporting limited impacts on social and motor skills in children with ASD (Date et al., 2024).

Given the lack of consensus regarding the duration of aquatic exercise intervention based on Halliwick’s method for children with autism, this study aims to evaluate the impact of a 12-week aquatic exercise regimen on social skills, motor skills, and balance in children with autism, as well as assess the durability of the intervention effects 4 weeks post-intervention cessation. Unique to this investigation is the integration of sensory integration assessment (CSIS) to elucidate the pathways of improvement. We hypothesize that 12 weeks of intervention will yield significant, sustainable gains in both motor and behavioral domains, persisting for at least 4 weeks post-intervention.

## 2 Methods

Due to the intensive nature of the 1:1 Halliwick intervention and the exploratory objective of evaluating multi-dimensional outcomes, this research was designed as a pilot study with four observation points: The initial assessment at week 0 served as the baseline evaluation (T0), with the mid-term evaluation at week 6 (T1), and end of intervention evaluation at week 12 (T2). The follow-up assessment was conducted 4 weeks post-intervention (T3).

### 2.1 Participants

This study was conducted from April 2023 to June 2023. The study was approved by the Medical Ethics Review Committee of Guangxi University (ethics number: 2023-018) and adhered to the principles outlined in the Declaration of Helsinki. Parents or caregivers of all children were informed about the study protocol and signed an informed consent form.

The inclusion criteria were as follows: (1) children aged 6–8 years old; (2) a mild to moderate ASD diagnosis from physicians or psychologists based on the Diagnostic and Statistical Manual of Mental Disorders, 5th edition; (3) the ability to follow instructions from researchers; (4) ability to perform requested physical intervention and executive function measures with the assistance of the research staff; and (5) absence of regular participation in any physical exercise outside of school physical education for at least 3 months before the study. Exclusion criteria were as follows: (1) presence of other chronic diseases; (2) physical disabilities precluding basic physical activities; and (3) infectious diseases (such as hepatitis, dysentery, conjunctivitis, skin diseases, and tuberculosis, etc.).

Regarding the sample size, since this is a pilot study, no formal sample size calculation was conducted. Therefore, this study does not meet the necessary conditions for conducting a clear hypothesis test.

**Figure 1:**
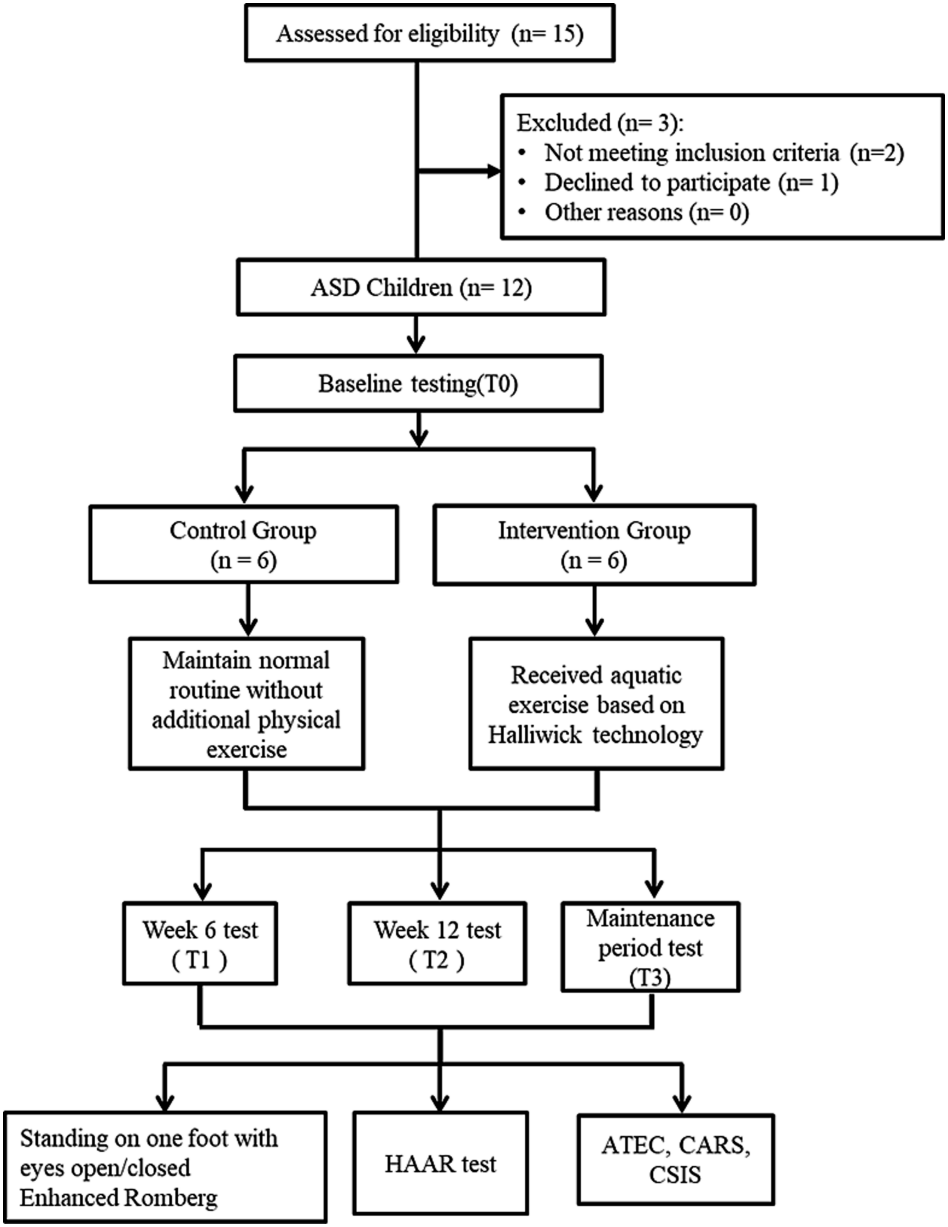
Flowchart of participant recruitment, group allocation, and assessment time points. T0 = Baseline; T1 = Week 6; T2 = Week 12; T3 = 4-week maintenance follow-up. ATEC, Autism Treatment Evaluation Checklist; CARS, Childhood Autism Rating Scale; CSIS, Child Sensory Integration Scale; HAAR, Humphries Assessment of Aquatic Readiness.

The recruitment and contact processes have been strictly standardized regardless of their gender or age. The initial contact with parents was conducted through a unified promotional brochure and standardized orientation sessions held at the rehabilitation center. A total of 15 children with ASD were initially screened for eligibility from the Guangxi Rehabilitation Center for Disabled Children with ASD. After excluding three candidates who did not meet the inclusion criteria or declined to participate, 12 male participants were successfully enrolled. The participates were only male because in the local clinical population, the incidence of autism spectrum disorders is higher in males, and during the recruitment process, all eligible volunteers were male. Participants were randomly assigned to either the control group (*n* = 6) or the intervention group (*n* = 6) at a 1:1 ratio using simple randomization via a random number table. The baseline consistency of all clinical and demographic variables was evaluated. All participants completed the 12-week intervention and the 4-week follow-up assessment. The detailed flow of participant recruitment, group allocation, and the experimental procedure is illustrated in Figure 1.

A third-party researcher who was not involved in the recruitment or intervention process generated the random allocation sequence. To ensure the concealment of the allocation, the group information was placed in sequentially numbered, opaque and sealed envelopes, and was only opened by the study coordinator after the baseline test was completed. Due to the nature of the underwater intervention, this study was unable to implement blinding for the children and their parents; however, except for HAAR assessment, all result evaluators were unaware of the group allocation during the intervention and follow-up assessment periods to ensure the objectivity. The HAAR assessment was conducted by a principal investigator and two independent observers. The two independent observers played a role in minimizing potential detection bias and ensuring the objectivity of the scores, thereby guaranteeing the overall reliability of the assessment. Demographic data for both groups are detailed in Table 1.

**Table 1:** Comparison of baseline characteristics between the two groups.

Group	Gender (male/female)	Age (year)	Height (cm)	Body weight (kg)
Control (*n* = 6)	6/0	7.17 ± 0.40	119.85 ± 2.35	24.80 ± 3.22
Intervention (*n* = 6)	6/0	7.25 ± 0.41	122.00 ± 7.21	21.97 ± 3.24

### 2.2 Intervention

The intervention group participated in aquatic interventions conducted in a swimming pool measuring 15 meters by 25 meters, with a water depth ranging from 1.1 to 1.5 meters and a temperature maintained at approximately 27 °C ± 1 °C. The intervention group received 1:1 guidance on Halliwick’s technical water intervention by an instructor for 60–70 min, three times a week, for a total of 12 weeks. The participants were divided into two groups and the intervention took place from 6:00 to 7:00 p.m. and from 7:20 to 8:20 p.m. The coaches were trained in Halliwick method and had experience teaching children with special needs to swim. Activities included warm-up exercises (5 min), land small muscle group and balance exercises (5 min), land leg exercises (10 min), and water equipment wearing (2 min), Halliwick’s “ten-steps procedure” (35 min), and end of course stretch relaxation (5–10 min) (Table 2) (Rohn et al., 2021). During the first 6 weeks, the intervention focused on mental adjustment and lateral rotation control; from weeks 7 to 12, it shifted to vertical balance and independent swimming skills. The content of the class is adjusted according to the acceptance level and learning speed of the participants, while the overall framework and learning sequence remain unchanged. The 12-week aquatic intervention program consists of 36 structured sessions. The detailed curriculum outline is presented in Supplementary Table S1. The coaches also used standardized tracking templates to record the completion status of the current class, the course plan, and any progress made of each participant (Supplementary Table S2). To ensure the consistency of the intervention, all coaches hold a case meeting once a week to record the progress of each child and cross-reference it with the official Halliwick manual. The control group received no exercise intervention and was instructed to maintain their usual routines without additional physical exercise. Parents of the control group were required to maintain a weekly activity log to ensure no additional organized physical exercise was introduced during the study period. After the study, control group participants were taught how to swim to recognize their contribution as controls.

**Table 2:** Main contents of the course.

Time	Main contents	Corresponds to the “10-steps program”
Week 1–2	Land and water breathing exercises Hold your breath in water Spinning and floating in the water	Point 1–5
Week 3–4	Floating exercise Land and water freestyle leg continuous movement exercises Land and water breaststroke leg exercises	Point 6–9
Week 5–6	Land, water breaststroke leg movement complete practice Land and water breaststroke practice	Point 9
Week 7–8	Land, water breaststroke hand movement complete practice Land breaststroke with complete motion breakdown exercise	Point 10
Week 9–10	Land and water breaststroke with complete movement practice Land freestyle hand movement break down exercise Ascent exercise	Point 10
Week 11–12	Land, water freestyle break down water practice Water freestyle with movement practice	Point 10

### 2.3 Follow-up

Four weeks after the intervention, assessments were conducted on both groups to evaluate the duration of the intervention effects.

### 2.4 Outcome measures

According to the aquatic intervention exercise plan, the intervention was divided into two stages: 0–6 weeks (first stage) and 7–12 weeks (second stage). Outcome measures were collected at all four time points (T0, T1, T2, and T3). Standardized scales, including the Autism Treatment Evaluation Checklist (ATEC), the Childhood Autism Rating Scale (CARS), and the Child Sensory Integration Scale (CSIS), were completed by parents or guardians who had lived with the child for at least 2 weeks, while physical assessments (HAAR, Balance ability) were conducted by blinded professional evaluators and the principal investigator.

The following measurement tools were employed to assess outcomes:

1. *Autism Treatment Evaluation Checklist (ATEC)*: Primarily used to evaluate the effect of intervention, aiding in understanding changes in the physical and mental health of autistic children more comprehensively, and is convenient for guardians to use (Jagadeesan et al., 2022; Rimland and Edelson, 1999). It consists of four components: language communication, social ability, cognitive ability, and health behavior (Ryan and Gianneschi, 2025).
2. *Children’s Autism Assessment Scale (CARS)*: Comprises 15 items assessing various domains such as interpersonal relationships, imitation, motor ability, adaptability to environmental changes, visual response. Each item was rated on a scale of 1 to 4, with higher scores indicating more severe autism symptoms (Stevanovic et al., 2021).
3. *Child Sensory Integration Scale (CSIS)*: The scale consists of 58 items rated on a five-point scale: “never,” “rarely,” “sometimes,” “often,” “and always,” where lower scores signify more severe sensory integration disorders (Ren et al., 1994; Li et al., 2023).
4. *Balance Ability Tests*:

Each balance test was conducted three times, and the result with the longest duration was taken as the final score. A 1-min break was given between the two tests to eliminate the influence of fatigue.

- Standing on one foot with eyes open or closed: During the test, participants stood alternately on one foot, with eyes open or closed, while the opposite foot was lifted off the ground with the knees bent and positioned against the knee of the standing leg (Capio et al., 2018).
- Enhanced Romberg: Participants stood with feet in front and behind without bending the knee, and the tip of the hind foot connected with the heel of the other foot. Once stable, timing began and continued until the participants moved either foot or lost balance (Deng et al., 2023).

1. *Humphries Assessment of Aquatic Readiness (HAAR)*: This scale was designed by Martin (1981) according to Halliwick method to evaluate participants’ aquatic motor skills, demonstrating high reliability and validity (above 0.90) (Travers et al., 2013). The HAAR assessment encompasses five stages: psychological adjustment, water entry, rotation, balance control, and independent movement in water (Shariat et al., 2024). To ensure the reliability of the HAAR assessment, the inter-rater agreement (IOA) was calculated in the study. Two trained independent observers (blinded to group allocation) and the principal investigator (not blinded) independently rated the performance of each participant simultaneously. The consistency was calculated using the point-by-point consistency method: the number of consistent ratings divided by the sum of consistent and inconsistent ratings, then multiplied by 100 (Pan, 2010). This calculation was performed separately for each stage of HAAR to verify the consistency of the rating process.
2. *Qualitative study*: Moreover, to complement the results of the quantitative study, we conducted semi-structured interviews with the six guardians of the intervention group at T1, T2, and T3, using a guide consisting of 11 questions (see Supplementary Table S3). Each interview lasted approximately 20 min. The credibility of the data was ensured through member checking, where the guardians verified the summaries. The interviews and subsequent thematic analysis were carried out by two sports rehabilitation experts from the research team. Both of them hold degrees in sports science and have over 3 years of experience in implementing sports interventions for children with ASD.

### 2.5 Statistical analysis

SPSS 27.0 statistical software was used for data analysis. Data normality was assessed using the Shapiro–Wilk test. We assessed the data for normality, and non-normally distributed data underwent logarithmic transformation to achieve normality. Independent sample t-tests were used to compare baseline characteristics among groups. To evaluate the intervention effects across four time points (T0, T1, T2, and T3), a repeated measures analysis of variance was conducted, with Group (Intervention vs. Control) as the between-subjects factor and Time as the within-subjects factor. Mauchly’s test of sphericity was applied; in cases where the assumption of sphericity was violated, the Greenhouse–Geisser correction was used to adjust the degrees of freedom. Beyond *p*-values, effect sizes were quantified using Partial Eta Squared (η^2^_p_), interpreted as small (0.01), medium (0.06), or large (0.14). For the *post hoc* pairwise comparisons, the Bonferroni correction method was employed. All data were expressed as mean ± standard deviation (SD), with *p* < 0.05 indicating significant differences and *p* < 0.01 indicating very significant differences.

### 2.6 Qualitative data analysis

Qualitative data analysis followed the six-stage inductive thematic analysis framework (Braun and Clarke, 2006). Firstly, two researchers independently reviewed the summaries of interviews. During the coding process, initial codes were generated based on recurring behavioral observations and guardians’ feedback. These codes were then classified into sub-themes and further refined into final overall themes. To ensure the rigor of the analysis, two sports rehabilitation experts independently completed the coding work and resolved any differences through consultation. A total of 54 important behavioral/feedback statements were extracted. Among them, 25 statements supported Theme 1 (improvement of daily biological rhythms), mainly involving sleep and eating habits. The remaining 29 statements supported Theme 2 (emotional and behavioral regulation), covering hyperactivity, stereotypical behaviors, and functional independence, etc.

## 3 Results

### 3.1 Baseline characteristics

Table 1 shows the characteristics of the study participants. Baseline characteristics were comparable between the two groups, with no statistically significant differences in any of the outcome measures before the treatment period (Tables 3–7).

**Table 3:** Comparison of CARS scores between the two groups.

Group	T0	T1	T2	T3	Source	*F*	*p*	η^2^_p_
Control (*n* = 6)	34.00 ± 2.75	33.00 ± 1.78	32.83 ± 1.16	32.17 ± 0.75	Time	12.165	0.002	0.549
Intervention (*n* = 6)	33.83 ± 2.04	31.17 ± 2.48^#^	30.00 ± 2.36^▲#^	30.00 ± 2.36^#^	Group	2.964	0.116	0.229
					Time × Group	2.447	0.142	0.197

T0: at baseline, T1: after 6 weeks, T2: after 12 weeks, T3: Follow up; CARS: Childhood Autism Rating Scale.

Repeated-measures ANOVA was performed. All data are presented as mean ± SD. Comparison between groups: ▲*p* < 0.05, ▲▲*p* < 0.01; Within-group comparisons: ^#^*p* < 0.05, ^##^*p* < 0.01.

**Table 4:** Comparison of ATEC scores between the two groups.

Items	Group	T0	T1	T2	T3	Source	*F*	*p*	η^2^_p_
Speech/Language/Communication	Control (*n* = 6)	13.504.80	13.83 ± 3.06	13.83 ± 3.06	13.50 ± 3.93	Time	0.476	0.702	0.045
	Intervention (*n* = 6)	9.50 ± 2.95	9.17 ± 3.43^▲^	8.17 ± 3.65^▲^	7.67 ± 3.88^▲^	Group	7.970	0.018	0.444
						Time × Group	0.453	0.717	0.043
Sociability	Control (*n* = 6)	12.33 ± 3.61	12.50 ± 2.51	11.67 ± 2.33	13.83 ± 2.85	Time	0.212	0.887	0.021
	Intervention (*n* = 6)	13.00 ± 4.73	13.17 ± 4.07	12.33 ± 5.82	12.17 ± 6.33	Group	0.002	0.965	0.000
						Time × Group	0.376	0.771	0.036
Sensory	Control (*n* = 6)	11.50 ± 4.32	11.17 ± 3.54	12.67 ± 3.50	13.50 ± 3.72	Time	0.154	0.790	0.015
	Intervention (*n* = 6)	11.00 ± 3.52	12.50 ± 4.13	11.00 ± 3.63	9.00 ± 3.16	Group	0.185	0.676	0.018
						Time × Group	0.782	0.437	0.073
Health/Physical/Behavior	Control (*n* = 6)	14.33 ± 6.43	16.00 ± 5.02	15.33 ± 3.88	13.00 ± 3.89	Time	0.956	0.426	0.087
	Intervention (*n* = 6)	15.50 ± 3.72	9.83 ± 2.99^▲##^	12.33 ± 4.76	11.17 ± 1.41	Group	0.633	0.445	0.060
						Time × Group	3.174	0.038	0.241
ATEC total	Control (*n* = 6)	51.67 ± 4.41	53.50 ± 4.76	53.50 ± 4.32	53.83 ± 2.63	Time	0.169	0.819	0.017
	Intervention (*n* = 6)	49.00 ± 5.40	44.67 ± 5.42^▲^	43.83 ± 12.79	45.00 ± 14.9	Group	4.525	0.059	0.312
						Time × Group	0.887	0.417	0.081

T0: at baseline, T1: after 6 weeks, T2: after 12 weeks, T3: Follow up; ATEC: Autism Treatment Evaluation Checklist.

Repeated-measures ANOVA was performed. All data are presented as mean ± SD. Comparison between groups: ▲*p* < 0.05, ▲▲*p* < 0.01; Within-group comparisons: ^#^*p* < 0.05, ^##^*p* < 0.01.

**Table 5:** Comparison of CSIS scores between the two groups.

Items	Group	T0	T1	T2	T3	Source	*F*	*p*	η^2^_p_
Vestibular imbalance interoceptive	Control (*n* = 6)	41.33 ± 3.26	39.67 ± 2.87	39.33 ± 3.50	39.83 ± 3.43	Time	4.367	0.011	0.304
	Intervention (*n* = 6)	44.00 ± 3.22	49.50 ± 9.13^▲^	55.33 ± 9.37^▲▲##^	46.17 ± 7.70	Group	8.694	0.015	0.465
						Time × Group	7.751	0.001	0.437
Defense of touch	Control (*n* = 6)	76.00 ± 6.51	69.67 ± 4.54	70.67 ± 4.08	72.50 ± 3.08	Time	0.586	0.532	0.055
	Intervention (*n* = 6)	73.67 ± 3.55	77.17 ± 9.10	82.00 ± 12.31	75.75 ± 13.115	Group	2.560	0.141	0.204
						Time × Group	3.027	0.086	0.232
Proprioceptive disorders	Control (*n* = 6)	34.17 ± 3.25	33.83 ± 3.71	35.17 ± 3.71	37.50 ± 3.78	Time	6.190	0.009	0.382
	Intervention (*n* = 6)	38.00 ± 4.14	41.17 ± 6.73^▲^	48.50 ± 9.73^▲#^	43.83 ± 5.19^▲^	Group	9.318	0.012	0.482
						Time × Group	3.495	0.053	0.259
Insufficient development of learning ability	Control (*n* = 6)	17.83 ± 3.06	18.17 ± 2.56	18.50 ± 2.16	19.00 ± 2.09	Time	9.873	0.003	0.497
	Intervention (*n* = 6)	16.17 ± 1.94	22.50 ± 7.00^#^	24.83 ± 3.54^▲▲##^	25.00 ± 2.82^▲▲##^	Group	5.583	0.040	0.358
						Time × Group	6.537	0.013	0.395

T0: at baseline, T1: after 6 weeks, T2: after 12 weeks, T3: Follow up; CSIS: Sensory Integration rating scale for children.

Repeated-measures ANOVA was performed. All data are presented as mean ± SD. Comparison between groups: ▲*p* < 0.05, ▲▲*p* < 0.01; Within-group comparisons: ^#^*p* < 0.05, ^##^*p* < 0.01.

**Table 6:** Comparison of balance ability test results between the two groups.

Items	Group	T0	T1	T2	T3	Source	*F*	*p*	η^2^_p_
Standing on left foot with eyes open (s)	Control (*n* = 6)	3.49 ± 0.88	4.68 ± 1.18	3.92 ± 0.92	4.41 ± 0.76	Time	32.381	0.001	0.764
	Intervention (*n* = 6)	3.52 ± 1.55	14.34 ± 5.45^▲▲##^	20.36 ± 6.75^▲▲##^	17.92 ± 5.29^▲▲##^	Group	30.952	0.001	0.756
						Time × Group	27.586	0.001	0.734
Standing on right foot with eyes open (s)	Control (*n* = 6)	3.10 ± 0.41	4.37 ± 2.08	3.80 ± 0.87	4.12 ± 1.72	Time	35.436	0.001	0.780
	Intervention (*n* = 6)	3.29 ± 0.90	15.49 ± 4.06^▲▲##^	20.37 ± 6.13^▲▲##^	18.63 ± 4.85^▲▲##^	Group	50.739	0.001	0.835
						Time × Group	28.440	0.001	0.740
Standing on left foot with eyes closed (s)	Control (*n* = 6)	2.09 ± 0.51	3.11 ± 1.37	2.61 ± 1.16	2.64 ± 0.93	Time	20.600	0.001	0.673
	Intervention (*n* = 6)	2.73 ± 0.93	6.26 ± 1.63^▲▲##^	10.75 ± 4.00^▲▲##^	8.08 ± 3.16^▲▲##^	Group	19.768	0.001	0.664
						Time × Group	16.522	0.001	0.623
Standing on right foot with eyes closed (s)	Control (*n* = 6)	2.09 ± 0.60	2.26 ± 1.22	1.69 ± 0.58	2.19 ± 0.68	Time	20.963	0.001	0.677
	Intervention (*n* = 6)	2.09 ± 0.56	6.76 ± 0.86^▲▲##^	9.69 ± 2.97^▲▲##^	7.12 ± 2.27^▲▲##^	Group	46.521	0.001	0.823
						Time × Group	24.513	0.001	0.710
Enhanced Romberg test(s)	Control (*n* = 6)	4.01 ± 0.76	4.49 ± 0.31	3.69 ± 0.12	3.54 ± 0.39	Time	83.161	0.001	0.893
	Intervention (*n* = 6)	4.42 ± 0.34	10.05 ± 1.16^▲▲##^	14.80 ± 1.68^▲▲##^	11.81 ± 0.94^▲▲##^	Group	332.029	0.001	0.871
						Time × Group	123.363	0.001	0.925

T0: at baseline, T1: after 6 weeks, T2: after 12 weeks, T3: Follow up.

Repeated-measures ANOVA was performed. All data are presented as mean ± SD. Comparison between groups: ▲*p* < 0.05, ▲▲*p* < 0.01; Within-group comparisons: ^#^*p* < 0.05, ^##^*p* < 0.01.

**Table 7:** Comparison of HAAR scores between the two groups.

Items	Group	T0	T1	T2	T3	Source	*F*	*p*	η^2^_p_
Mental adjustment	Control (*n* = 6)	70.00 ± 24.49	66.67 ± 27.32	70 ± 24.49	66.66 ± 27.32	Time	6.980	0.004	0.411
	Intervention (*n* = 6)	70.00 ± 10.95	90 ± 16.733^##^	100 ± 0.00^▲##^	89.44 ± 11.62^##^	Group	3.322	0.098	0.249
						Time × Group	8.196	0.002	0.450
Introduction to water environment	Control (*n* = 6)	25.00 ± 15.16	26.67 ± 13.66	23.33 ± 15.05	25.00 ± 13.78	Time	18.386	0.001	0.648
	Intervention (*n* = 6)	26.67 ± 17.51	31.67 ± 24.01	93.33 ± 12.11^▲▲##^	89.44 ± 17.18^▲▲##^	Group	25.356	0.001	0.717
						Time × Group	19.740	0.001	0.664
Rotations	Control (*n* = 6)	0	0	0	0	Time	7.083	0.027	0.415
	Intervention (*n* = 6)	0	22.22 ± 27.21^#^	44.44 ± 40.36^▲▲##^	32.40 ± 29.89^▲▲##^	Group	12.250	0.006	0.551
						Time × Group	7.083	0.027	0.415
Balance and control	Control (*n* = 6)	20.83 ± 10.20	22.91 ± 12.28	22.91 ± 9.409	20.83 ± 10.20	Time	11.164	0.001	0.528
	Intervention (*n* = 6)	20.83 ± 6.45	58.33 ± 18.81^▲▲##^	89.58 ± 14.61^▲▲##^	70.485 ± 10.57^▲▲##^	Group	18.171	0.002	0.645
						Time × Group	11.425	0.001	0.533
Independent movement in water	Control (*n* = 6)	0	0	0	0	Time	50.660	0.001	0.835
	Intervention (*n* = 6)	2.77 ± 6.80	44.42 ± 25.08^▲▲##^	78.6 ± 38.44^▲▲##^	58.31 ± 20.40^▲▲##^	Group	75.568	0.001	0.883
						Time × Group	50.660	0.001	0.835

T0: at baseline, T1: after 6 weeks, T2: after 12 weeks, T3: Follow up; HAAR: Humphries’ Assessment of Aquatic Readiness.

Repeated-measures ANOVA was performed. All data are presented as mean ± SD. Comparison between groups: ▲*p* < 0.05, ▲▲*p* < 0.01; Within-group comparisons: ^#^*p* < 0.05, ^##^*p* < 0.01.

### 3.2 Adherence and control group monitoring

Based on the analysis of the weekly activity logs of the control group, all 6 participants strictly adhered to the research protocol. During the 12-week intervention period, none of them participated in any structured hydrotherapy or additional physical therapy programs beyond their standard daily school activities.

### 3.3 CARS

After the exercise intervention, there was a temporal effect on the CARS score in the two groups (*F* = 12.165, *p* = 0.002, η^2^_p_ = 0.549). CARS scores at T1, T2, and T3 were significantly lower in the intervention group at T0 (*p* < 0.05), whereas no significant changes were observed in the control group (*p* > 0.05). Notably, CARS scores in the intervention group at T2 were significantly lower than those in the control group (*p* < 0.05) (Table 3). These findings indicate that the aquatic exercise based on Halliwick method significantly reduced symptoms of autism after six and 12 weeks, with the effect persisting for at least 4 weeks.

### 3.4 ATEC

According to Cohen’s criteria, the Physical/Health/Behavior subscale score demonstrated a large effect size (η^2^_p_ = 0.241), and the ATEC total score also showed a medium effect (η^2^_p_ = 0.081). This indicates that despite the limited sample size leading to insufficient statistical power, the underwater exercise intervention still showed a clinically relevant trend of symptom improvement in the experimental group. Simple effect analysis revealed that the Physical/Health/Behavior score of the experimental group significantly decreased at T1 compared to T0 (*p* < 0.01), while no significant change was observed in the control group. Although a significant group effect was found in the Language/Communication dimension (*F* = 7.970, *p* = 0.018, η^2^_p_ = 0.444), there was no significant interaction (*p* = 0.717) (Table 4). This suggests that while children in the aquatic exercise group maintained superior verbal performance compared to the control group, the rate of improvement did not statistically differ between groups. These preliminary results indicate that Halliwick-based aquatic exercise holds promise for enhancing health behaviors and maintaining verbal communication skills in children with ASD.

### 3.5 CSIS

After 12 weeks, in the assessment of sensory integration ability, repeated measures ANOVA revealed that the time × group interaction for vestibular perception (*F* = 7.751, *p* = 0.001, η^2^_p_ = 0.437) and learning ability (*F* = 6.537, *p* = 0.013, η^2^_p_ = 0.395) were both highly significant. Although the interaction for proprioception was on the verge of statistical significance (*F* = 3.495, *p* = 0.053), it showed a large effect size (η^2^_p_ = 0.259), suggesting a trend of substantial clinical significance for this intervention. In the intervention group, scores for vestibular sensation and proprioception significantly increased at T2 compared to T0 (*p* < 0.01 and *p* < 0.05). Learning ability scores revealed significant increases at T1, T2, and T3 (*p* < 0.05, *p* < 0.01, and *p* < 0.01). The control group did not exhibit significant score changes compared with the baseline (*p* > 0.05). The intervention group also had significantly higher vestibular sensation scores than the control group at T1 and T2 (*p* < 0.05 and *p* < 0.01), higher proprioception scores at T1, T2, and T3 (*p* < 0.05), and higher learning ability scores at T2 and T3 (*p* < 0.01) (Table 5). Thus, six and twelve weeks of Halliwick-based aquatic exercise significantly improved learning, proprioception, and vestibular function in children with autism, with effects lasting for 4 weeks.

### 3.6 Balance

After 12 weeks of intervention, all balance indicators (single-leg standing with eyes open/closed, enhanced Romberg test) showed a highly significant time × group interaction (all *p* < 0.01), accompanied by a large effect size (η^2^_p_ ranging from 0.623 to 0.925) (Table 6). This indicates that over 60% of the variation in balance ability can be explained by the intervention measures, demonstrating the good clinical effectiveness of underwater movement based on the Halliwick method in improving the postural control ability of children with autism. The simple effect analysis showed the scores of the intervention group in all aspects at T1, T2, and the follow-up period T3 were significantly improved compared to T0 (*p* < 0.01), while the control group showed no significant improvement at any time point. The inter-group comparison further confirmed that the balanced performance of the experimental group in each stage after the intervention was superior to that of the control group (*p* < 0.01) (Table 6). Thus, 6 and 12 weeks of aquatic exercise based on Halliwick method significantly improved balance, with effects lasting for 4 weeks.

### 3.7 HAAR

In the HAAR aquatic rehabilitation assessment, the results of the repeated measures analysis of variance showed that all dimensions and the total score all demonstrated a highly significant time × group interaction (all *p* < 0.05) (Table 7). It is notable that underwater independent movement (*F* = 50.660, *p* = 0.001, η^2^_p_ = 0.835) and the total HAAR score (*F* = 45.921, *p* = 0.001, η^2^_p_ = 0.821) showed extremely large effect sizes. The simple effect analysis showed that from the T1 or T2 stage of the experiment, the scores of all HAAR dimensions in the experimental group were significantly higher than the baseline (T0) (*p* < 0.01). Among them, the rotation dimension showed a significant increase at T1 (*p* < 0.05), and the score of the underwater entry module continued to increase significantly at T2 and T3 (*p* < 0.01). The control group did not observe any significant changes in all indicators compared to the baseline throughout the experiment (*p* > 0.05). Further comparison between the groups revealed that the score of the psychological adjustment (Mental adjustment) dimension in the experimental group was significantly higher than that of the control group at T2 (*p* < 0.05). In the balance control and underwater independent movement dimensions, the scores of the experimental group were significantly higher than those of the control group at T1, T2, and the follow-up period T3 (*p* < 0.01). The group differences in the rotation dimension also reached an extremely significant level at T2 and T3 stages (*p* < 0.01). This suggests that six and twelve weeks of Halliwick-based aquatic exercise significantly improved motor skills in children with autism, with effects sustaining for 4 weeks.

### 3.8 Qualitative Observations from Guardian interviews

To gain preliminary insights into the perceived impact of the intervention, thematic analysis was performed on the semi-structured interviews with guardians.

Theme 1: Improvements in daily biological rhythms. Guardians frequently reported enhanced sleep quality and dietary behaviors. For instance, one parent noted: “He used to take hours to fall asleep, but after the pool days, he settles down faster than before,” suggesting a potential link between aquatic physical exertion and sleep-onset latency. Regarding diet, another guardian observed a reduction in food selectivity: “He became more willing to touch and try textures he previously avoided.”

Theme 2: Emotional and Behavioral Regulation. Guardians reported a reduction in hyperactive energy and improved emotional stability. As one guardian described: “There is a noticeable calm that lasts into the evening, making him more receptive to home routines.”

Summary. The benefits of the Halliwick-based aquatic exercise may extend beyond core autistic symptoms to broader aspects of life quality.

## 4 Discussion

Previous studies have shown that exercise intervention can effectively reduce the symptoms of autistic children (Sarabzadeh et al., 2019; Phung and Goldberg, 2021; Jagadeesan et al., 2022). However, few studies have comprehensively evaluated the effects of aquatic exercise on autistic symptoms, balance, and motor skills using effect size analysis. This study showed that 12 weeks of aquatic exercise based on Halliwick method improved balance and motor abilities, characterized by large effect sizes (η^2^_p_ up to 0.925). Furthermore, clinically relevant improvements were observed in ATEC language communication, health behaviors and total scores, alongside significant gains in proprioception, vestibular sensation, and learning ability. These multi-dimensional gains, particularly the robust effects on physical functioning, persisted for at least 4 weeks.

In this study, CARS scores in the intervention group were significantly lower than those in the control group at 6 weeks, 12 weeks and 4 weeks post-intervention. This finding indicates that benefits emerged early, continued throughout the program, and persisted after training ceased. This is consistent with the results of a 10-month swimming intervention in which total CARS scores were lower than those of the control group (Caputo et al., 2018). Notably, while previous long-term interventions, such as the 10-month program by Caputo et al. (2018) and the 4-month program by Xu et al. (2018), reported significant CARS reductions, our 12-week Halliwick-based program achieved comparable clinical outcomes in a significantly shorter duration. This high efficiency is further supported by the large effect size observed in this study (η^2^_p_ = 0.549), which underscores the robust impact of aquatic exercise on core autism symptoms. The early improvement at 6 weeks may be attributed to the ‘Mental Adjustment’ phase of the Halliwick method, which reduces sensory defensive behaviors and anxiety in aquatic environments. Furthermore, the sustained reduction in CARS scores aligns with the observed gains in proprioception and vestibular sensation (as measured by CSIS), suggesting that enhanced sensory integration serves as a foundational mechanism for broader behavioral improvements. Taken together, Halliwick-based aquatic exercise provides a highly efficient and sustainable intervention for reducing the symptom severity of children with ASD.

Social communication and verbal communication disorders are the two core symptoms of autism (Sharma et al., 2018). In this study, ATEC assessment showed significant decrease in language/communication scores among children in the swimming training, accompanied by a large effect size. The substantial effect size, coupled with baseline homogeneity, suggests a robust clinical trend toward improved verbal skills in the intervention group compared to the control group. This is consistent with previous showing that 8 weeks of structured exercise improves language and communication skills in people with autism (Ye et al., 2019). However, social communication scores did not show significant improvement, while other studies have shown that physical exercise can be an important option to improve the social skills of children with autism (Koh, 2024; Lopez-Diaz et al., 2021). This may be related to the one-to-one water teaching model adopted in this study, in which teachers consciously communicate with children using frequent verbal cues throughout class time. Conversely, the lack of peer-to-peer interaction and diverse social scenarios inherent in a one-to-one setting, coupled with the fact that skills acquired in a structured and predictable aquatic environment may not naturally generalize to complex and dynamic social contexts, may have limited the intervention’s impact on broader social communication skills. This suggests that while individual aquatic therapy is highly effective for targeting specific functional and verbal goals, supplementary group-based modules may be necessary. A phased transition model moving from 1:1 technical instruction to small-group inclusive settings could encourage children to apply their improved self-regulation and communication skills in more varied social scenarios, thereby effectively addressing multidimensional social-communication deficits.

Our quantitative analysis revealed that the health behavior module score and total ATEC score decreased with a notable Time × Group interaction. Qualitative feedback from guardians provided deeper insight into these quantitative gains: before the swimming training intervention, participants (e.g., P2 and P3) generally struggled with picky eating, and sleep onset latency. After participating in the aquatic exercise intervention, these domains were significantly improved after 6 weeks of intervention. At the initial stage, the caregivers mainly noticed an improvement in sleep efficiency; while by the end of the 12-week program, more complex changes in dietary variety and emotional stability also became evident. Specifically, the increased physical exertion attributable to the high resistance and buoyancy of the water environment likely regulated the children’s autonomic nervous system and energy expenditure, leading to the observed increase in nap frequency and better sleep quality. These findings align with the sensory integration hypothesis, where the intense tactile and vestibular stimulation of water helps normalize sensory processing, thereby reducing picky eating behaviors often rooted in oral sensory defensiveness. Compared with the studies by Ye et al. (2019) and Xu et al. (2018) on adolescents, this research has demonstrated that short-term (12-week) underwater intervention also has remarkable effectiveness in improving physiological regulation (such as digestion and sleep) in younger children with ASD.

Regarding balance, ASD patients often face challenges associated with sensory integration disorders and poor motor coordination, making them prone to poor postural control, unstable gait, and uncoordinated balance disorders (Uzunoğlu et al., 2025; Lim et al., 2019; Cham et al., 2021). Research has identified an increase in postural instability when ASD patients stand on one leg, indicating potential sensory impairments in balance control (Ben Hassen et al., 2023; Doumas et al., 2016; Chisari et al., 2024). In this study, compared with the control group, the intervention group experienced significant improvements in balance performance, including standing on one foot with eyes closed, enhanced Romberg, single-leg standing and Romberg tests—characterized by exceptionally large effect sizes (η^2^_p_ > 0.8). Consistent with the results of the other research, 12-week adaptive training can improve the balance of children with ASD, and this effect persisted 4 weeks post-intervention (Orhan et al., 2024).

Many children with autism have demonstrated successful motor skill acquisition in aquatic environments, largely due to the properties of water, such as buoyancy and resistance, which slow down body movements and reduce the effects of gravity (Marzouki et al., 2022; Salar et al., 2024). This unique property allows children to practice essential swimming and movement skills more effectively with fewer physical constraints. In this study, significant improvements were observed in HAAR scores at the sixth and twelfth training weeks. It is likely not merely a reflection of simple behavioral adaptation, and the sustained performance observed during the subsequent 4-week observation period indicates a certain degree of neurologically-based re-adjustment. Multisensory feedback, especially the continuous proprioceptive and vestibular stimulation provided by water resistance may enhance the ability of the central nervous system to integrate sensory input, thereby improving postural stability and motor control (Djordjević et al., 2022; Zhao et al., 2024).

Balance ability and motor proficiency depend on the integration of proprioceptive and vestibular inputs within the central nervous system (Ottenbacher and Short, 1985). Consistent with this mechanism, our CSIS results revealed notable improvements in learning, proprioceptive, and vestibular functions following the intervention. The constant multidirectional resistance of water provides continuous somatosensory feedback, effectively ‘recalibrating’ the vestibular system and enhancing neuromuscular coordination (Marzouki et al., 2022; Salar et al., 2024). Given the correlational nature of our data, these findings should be regarded as evidence that can help formulate hypotheses. Taken together, the findings of this study suggest that Halliwick-based aquatic exercise enhances motor performance in children with autism through multiple pathways, including improvements in sensory integration, postural balance, and neuromuscular coordination.

The significant improvements and large effect sizes observed in our study contrast sharply with the mixed results summarized by Date et al. (2024) in their recent systematic review. The differences can be explained by the following factors. Firstly, although Date et al. pointed out that many studies had issues with poor methodological quality and insufficient intervention density, our study implemented a structured 12-week intervention with consistent intervention frequency (three times per week), providing a higher treatment dose. Secondly, the selection of outcome indicators is crucial. Date et al. discussed the problem of lacking specific population targeting and reliable exercise scales; in contrast, we used the HAAR and CARS scales, which are specifically designed to capture aquatic functional progress and behavioral changes related to autism, potentially making them more sensitive to intervention effects than general balance tests. Finally, our sample is of a pilot nature, which may lead to more concentrated progress. Therefore, although our research results are encouraging, we agree with Date et al. (2024) that given the limited sample size, these large effect values should be interpreted with caution.

Given the significance of conducting follow-up studies on the effectiveness of many exercises intervention measures for children with autism, we included a follow-up assessment 4 weeks after completion of the 12-week swimming intervention to determine the persistence and sustainability of the results (Belaiba et al., 2024; Wang et al., 2022). After conducting a more detailed analysis of the data from T2 to T3, although a marginal regression in scores was observed at week 16 compared to the peak at week 12, the balance and motor skills-remained significantly superior to baseline levels (*p* < 0.05). This suggests that 12-week Halliwick-based aquatic exercise may induce lasting neuromuscular adaptations. The slight decline underscores that children with autism may require long-term personalized swimming programs to optimize functional maintenance and prevent skill attrition. Meanwhile, we also observed that the effectiveness of the intervention measures may also be influenced by the specific symptom characteristics of each participant. The children with a higher level of sensory defense at baseline may require a longer mental adjustment stage before being able to perform the propulsion action; while those with primary motor coordination problems showed more rapid progress in posture balance after achieving water adaptation. This highlights the necessity of establishing individualized progress rates in standardized water therapy programs. Overall, in order to determine the long-term sustainability of these effects, future research should increase the duration of the follow-up to 3 to 6 months to observe whether the benefits brought by underwater exercise can persist over a longer period without the need for continuous intervention measures.

Furthermore, the effectiveness of the Halliwick intervention method is significantly influenced by the specific symptom characteristics of the participants. The analysis of individual training records (Supplementary Table S2) indicates that children who had a stronger sense of defense at baseline (such as excessive sensitivity to touch) had a steeper learning curve during the initial “psychological adjustment” stage and required more time in contact with water to enter the advancement stage. Conversely, participants with a weaker response to vestibular input seemed to learn faster from the high-resistance water environment and made faster progress in posture balance. This suggests that the “treatment dose” and the progress speed should vary from person to person; although the 36 training unit schedule (Supplementary Table S1) provides a robust standardized framework, the speed of neuro-motor adaptation is essentially closely related to each child’s unique sensory processing characteristics.

This study has some limitations. First, although our results indicate beneficial effects of Halliwick-based aquatic exercise on children with autism, the sample size limited the statistical power to detect subtle interaction effects in certain ATEC domains, such as social communication. It should also be noted that the relatively large effect size observed may be due to the small sample size, which is easy to lead to a bias; therefore, these results should be regarded as preliminary indicators of the intervention effect rather than precise population statistics. Nevertheless, the large effect sizes reported here provide a critical foundation for future power calculations. Second, although standardized tools were used, the reliance on caregiver reports for ATEC and CSIS may introduce subjective bias. Third, due to the inherent nature of aquatic intervention, it was impossible to blind participants and instructors to group assignments. Similarly, during the HAAR assessment process, the involvement of the principal investigator may introduce potential biases. These factors may have introduced potential reporting bias in subjective assessments (e.g., ATEC and CARS). However, in order to mitigate these influences, we implemented the inter-rater agreement for HAAR and employed independent third-party professionals to conduct objective physical assessments (such as balance tests), thereby safeguarding the impartiality and reliability of the final data. Fourth, since the sample is entirely composed of young boys, it is necessary to further verify whether these research results can be applied to girls or older children with autism. Fifth, due to the absence of an active control group, it is difficult to distinguish the specific effects of the Halliwick method from the general benefits brought about by structured physical activities or social attention. Finally, future research should employ large-scale randomized controlled trials (RCTs) with extended longitudinal follow-ups and incorporate objective neurophysiological indicators (e.g., EEG or fNIRS) to clarify the cortical mechanisms underlying aquatic-induced motor and behavioral recovery.

## 5 Conclusion

This pilot study provides preliminary evidence of the feasibility and potential benefits of a Halliwick-based aquatic exercise program for children with autism spectrum disorder. The results showed that a 12-week intervention may help improve children’s social communication skills, sensory integration, balance, and hydrotherapy adaptability, with some effects lasting at least 4 weeks after the intervention ended. Given the limited sample size of this study, the results require careful interpretation. Future larger-scale randomized controlled trials are needed to validate these findings and clarify the long-term effects of Halliwick-based hydrotherapy interventions.

## Data Availability

The original contributions presented in the study are included in the article/Supplementary material, further inquiries can be directed to the corresponding author.
