# Supplementary material for: Effects of Halliwick-based aquatic exercise on social and motor skills of children with autism spectrum disorder: a pilot study
Source: Front Psychol. 2026 May 19;17:1832876. doi: 10.3389/fpsyg.2026.1832876 (PMC13226205; doi:10.3389/fpsyg.2026.1832876)
Supplement: Supplementary file 1 [file Data_Sheet_1.pdf]

Table S1 Detailed 36-Session Aquatic Exercise Intervention Program

Week 1, Session 1 Theme: Psychological Adaptation Phase<br>1. Emotional Regulation & Warm-up (5 mins)<br>Calming the participants and performing light stretching/joint mobilization.<br>2. Dry-land Exercises (10 mins)<br>•Finger blowing: 5 seconds × 30 reps (focused on controlled exhalation).<br>•Inhalation and breath-holding: 5 seconds × 30 reps.<br>Combined drill: 5 seconds breath-holding followed by 5 seconds finger blowing × 30 reps.<br>3. In-water Session (45 mins)<br>(1) Poolside Kicking (2 mins): Sitting on the edge and kicking the water surface.<br>(2) Water Adaptation & Surface Breathing (8 mins):<br>• Standing in the water while holding the pool edge.<br>• Blowing air onto the water surface.<br>• Exhaling into the water while standing.<br>(3) Shallow Water Immersion (10 mins):<br>•Sitting on the shallow pool floor.<br>•Blowing ping-pong balls on the water surface.<br>•Exhaling into the water from a seated position.<br>(4) Mobility & Coordination (10 mins):<br>•Walking and vertical jumping in the water.<br>•Blowing ping-pong balls while stationary and while moving.<br>(5) Submersion & Breath-holding (15 mins):<br>•Standing and holding the pool edge with the face touching the water surface for breath-holding.<br>•Breath-holding while fully standing in water.<br>Breath-holding while sitting with the face submerged.<br>4. Post-Session Free Play & Consultation (10 mins)<br>•Unstructured free play for the participants.<br>•Communication between the instructor, parents, and participants to assess the session's impact.<br>5. Homework Assignment<br>•Breathing Practice: Inhalation and exhalation drills.<br>•Freestyle Leg Kicks: 300 repetitions total (at least 30 reps per set).
Week 1, Session 2 Theme: Rotation Control I & Simple Progression I (Freestyle Leg Kicks)<br>1. Emotional Regulation & Warm-up (5 mins): Calming exercises and joint mobilization.<br>2. Dry-land Exercises (10 mins):<br>• Finger blowing: 5s × 20 reps.<br>• Inhalation & breath-holding: 5s × 20 reps.<br>• Combined drill (5s breath-holding + 5s finger blowing): × 20 reps.<br>• Freestyle leg kick drills (deconstructed): 30 reps × 5 sets.<br>3. In-water Session (45 mins):<br>(1) Basic Drills:<br>• Inhalation & exhalation holding the pool edge: × 30 reps.

• Inhalation & breath-holding: 5s × 30 reps.

• Freestyle leg kick practice.

(2) Phase I: Sagittal Rotation Control (20 mins):

• Lateral neck flexion in a vertical position to submerge ears.

• Lateral movement in a vertical position.

• Lateral trunk stretching in a seated position.

• Submerging the head while stationary.

(3) Phase II: Transversal Rotation Control (20 mins):

• Blowing bubbles in a vertical position (head down).

• Transitioning from vertical to prone floating.

• Transitioning from vertical to supine floating.

• Recovering from prone/supine floating back to a vertical position.

4. Post-Session Free Play & Consultation (10 mins):

Communication with parents and participants regarding session feedback.

5. Homework Assignment:

• Breathing practice (inhalation/exhalation).

• Freestyle leg kicks: 300 repetitions total (minimum 30 reps per set).

Week 1, Session 3 Theme: Rotation Control II & Simple Progression I (Freestyle Leg Kicks)

1. Warm-up & Joint Mobilization (5 mins): General stretching and joint flexibility exercises.

2. Dry-land Exercises (10 mins):

• Breaststroke leg kick drills (kneeling position) integrated with breathing practice.

• Continuous freestyle leg kicks: 30 reps × 10 sets.

3. In-water Session (45 mins):

(1) Phase III: Longitudinal Rotation Control

• Spinning in place in a vertical position.

• Prone floating with the face submerged.

• Transitioning from a prone position to a supine (face-up) floating position.

(2) Phase IV: Combined Rotation Control

• Sequence: From a seated position → standing → prone floating → supine floating.

• Sequence: From standing → supine floating → prone floating → standing → seated.

• Freestyle leg kick practice (integrated with propulsion).

4. Post-Session Free Play & Consultation (10 mins): Communication with parents and participants regarding progress and feedback.

5. Homework Assignment

• Breathing practice (inhalation and exhalation).

• Freestyle leg kicks: 300 repetitions total (minimum 50 reps per set).

Week 2, Session 1 (Theme: Floating (Static Balance & Gliding in Turbulence I) + Simple Progression I (Freestyle Leg Kicks))

1. Warm-up & Joint Mobilization (5 mins): General stretching and joint flexibility exercises.

2. Dry-land Exercises (10 mins)

• Inhalation & breath-holding: 5s × 30 reps × 2 sets.

• Toe towel curls (intrinsic foot muscle exercise): 25 reps × 3 sets.

• Continuous freestyle leg kick drills: 30 reps × 5 sets.<br>3. In-water Session (45 mins)<br>(1) Phase I: Floating and Balance Training<br>• Assisted floating: Teacher supports the waist while feet leave the pool floor (5 mins).<br>• Assisted floating with breath-holding: Teacher supports the waist while feet leave the pool floor (5 mins).<br>• Hand-held floating: Teacher holds participant's hands while feet leave the floor (5 mins).<br>• Hand-held floating with breath-holding (5 mins).<br>• Independent floating: Lifting feet off the floor stationary and independently (5 mins).<br>• Wall push-off (Gliding) practice (5 mins).<br>(2) Propulsion Drills: Continuous freestyle leg kick practice.<br>4. Post-Session Free Play & Consultation (10 mins): Unstructured water time and feedback exchange with parents/participants.<br>5. Homework Assignment<br>• Breathing practice (inhalation and exhalation).<br>• Freestyle leg kicks: 300 repetitions total (minimum 100 reps per set).
Week 2, Session 2 Theme: Floating (Static Balance & Gliding in Turbulence II) + Simple Progression I (Freestyle Leg Kicks)<br>1. Warm-up & Joint Mobilization (5 mins): Stretching and joint flexibility exercises.<br>2. Dry-land Exercises (15 mins)<br>• Breaststroke drills: Kneeling breaststroke leg kicks integrated with 5s breath-holding × 15 reps × 2 sets.<br>• Lower limb micro-muscle training: Toe towel curls × 30 reps × 3 sets.<br>• Balance training: Single-leg standing balance practice.<br>• Continuous freestyle kicks: 100 reps × 2 sets.<br>• Breaststroke leg kicks: Deconstructed movement practice × 10 reps × 10 sets.<br>3. In-water Session (40 mins)<br>Phase II: Advanced Floating and Gliding<br>• Statice Balance: Mushroom float (jellyfish float), stationary prone float, and stationary supine float.<br>• Gliding (Wall Push-offs): Prone glide from the wall (5 mins).<br>• Dynamic Drills: Supine glide from the wall; repeated prone and supine gliding sets (10 mins).<br>• Propulsion: Continuous freestyle leg kick practice.<br>4. Post-Session Free Play & Consultation (10 mins): Participant free play and instructor-parent progress review.<br>5. Homework Assignment<br>• Breathing: Inhalation and exhalation practice.<br>• Freestyle kicks: 300 repetitions total (min. 100 reps/set).<br>• Breaststroke kicks: 10 reps × 5 sets.
Week 2, Session 3Theme: Simple Progression I (Freestyle Leg Kicks)<br>1. Warm-up & Joint Mobilization (5 mins): Stretching and joint flexibility exercises.<br>2. Dry-land Exercises (10 mins):<br>• Breaststroke drills: Kneeling breaststroke leg kicks integrated with breathing practice.<br>• Endurance training: 200 continuous freestyle leg kicks.<br>• Skill acquisition: Deconstructed breaststroke leg kick instruction, 20 reps × 5 sets.<br>3. In-water Session (45 mins):

• Core Instruction: Intensive freestyle leg kick practice (focusing on propulsion efficiency and maintaining a streamlined body position).<br>4. Post-Session Free Play & Consultation (10 mins): Unstructured water time and communication with parents regarding the participant's performance.<br>5. Homework Assignment:<br>• Breathing: Inhalation and exhalation practice.<br>• Freestyle kicks: 300 repetitions total (minimum 100 reps per set).<br>• Breaststroke kicks: 10 reps × 5 sets.
Week 3, Session 1 Theme: Simple Progression II (Breaststroke Leg Kicks)<br>1. Warm-up & Endurance (5 mins):<br>• General stretching and joint mobilization.<br>• 200 continuous freestyle leg kicks.2. Dry-land Exercises (15 mins):<br>• Skill Acquisition: Deconstructed breaststroke leg kick practice, 30 reps × 3 sets.<br>• Coordination: Continuous breaststroke leg kick drills, 15 reps × 4 sets.<br>• Fine Motor & Balance: Toe towel curls × 30 reps × 3 sets; Single-leg standing balance practice.<br>3. In-water Session (40 mins):<br>• Warm-up Laps: Freestyle leg kicks, 25m × 4 laps.<br>• Semi-water Drills (Poolside): Deconstructed breaststroke leg kicks while leaning on the pool edge, 20 reps × 4 sets.<br>• Semi-water Coordination: Poolside breaststroke leg kick coordination (2 kicks per breath), 30 reps × 3 sets.<br>• In-water Drills: Holding the pool edge: Deconstructed breaststroke kicks with head above water, 30 reps × 3 sets.<br>•Holding the pool edge: Breaststroke kicks with breath-holding/rhythmic breathing (2 kicks per breath), 10 reps × 4 sets.<br>4. Post-Session Free Play & Consultation (10 mins): Unstructured water play and feedback session with parents.<br>5. Homework Assignment:<br>• Freestyle leg kicks: 300 reps total.<br>• Deconstructed breaststroke kicks: 30 reps × 5 sets.<br>• Continuous breaststroke kicks: 30 reps × 5 sets.
Week 3, Session 2 Theme: Simple Progression II (Breaststroke Leg Kicks)<br>1. Warm-up & Endurance (5 mins):<br>• General stretching and joint mobilization.<br>• 20 continuous freestyle leg kicks.<br>2. Dry-land Exercises (15 mins):<br>• Breathing Coordination: Kneeling breaststroke leg kicks with integrated breathing practice (2 mins).<br>• Skill Acquisition: Deconstructed breaststroke leg kick practice: 15 reps × 3 sets.<br>• Motor Control: Continuous breaststroke leg kick drills: 20 reps × 3 sets.<br>• Fine Motor Drills: Toe towel curls: 30 reps × 2 sets.<br>3. In-water Session (40 mins):<br>• Semi-water Drills (Poolside):<br>- Deconstructed breaststroke leg kicks (leaning on the edge): 20 reps × 4 sets.<br>- Full breaststroke leg kick cycle with coordination (2 kicks per breath): 30 reps × 3 sets.

• Warm-up Laps: Freestyle leg kicks: 6 laps.<br>• In-water Drills (Holding Edge):<br>- Stationary breaststroke leg kick practice: 20 reps × 5 sets.<br>- Stationary breaststroke leg kicks with rhythmic breathing (2 kicks per breath): 10 reps × 5 sets.<br>4. Post-Session Free Play & Consultation (10 mins): Participant free play and instructor-parent feedback.<br>5. Homework Assignment:<br>• Freestyle leg kicks: 300 reps total.<br>• Continuous breaststroke leg kicks: 30 reps × 5 sets.
Week 3, Session 3 Theme: Simple Progression II (Breaststroke Leg Kicks)<br>1. Warm-up & Endurance (5 mins):<br>• General stretching and joint mobilization.<br>• 200 continuous freestyle leg kicks.<br>2. Dry-land Exercises (15 mins):<br>• Motor Control: Continuous breaststroke leg kick drills, 15 reps × 4 sets.<br>• Repetitive Conditioning: Continuous breaststroke leg kicks, 20 reps × 5 sets.<br>• Fine Motor & Balance: Toe towel curls: 40 reps × 3 sets; Single-leg standing balance practice.<br>3. In-water Session (40 mins):<br>• Semi-water Coordination (Poolside): Breaststroke leg kick coordination with rhythmic breathing (3 kicks per breath): 30 reps × 3 sets.<br>• Endurance Laps: Freestyle leg kicks: 8 laps.<br>• Skill Consolidation: Intensive breaststroke leg kick practice (focusing on symmetrical kicking and gliding distance).<br>4. Post-Session Free Play & Consultation (5 mins): Brief unstructured water time and progress update with parents.<br>5. Homework Assignment:<br>• Freestyle leg kicks: 300 reps total.<br>• Continuous breaststroke leg kicks: 30 reps × 5 sets.
Week 4, Session 1 Theme: Simple Progression II (Breaststroke Leg Kicks & Gliding)<br>1. Warm-up & Endurance (15 mins):<br>• General stretching and joint mobilization.<br>• 200 continuous freestyle leg kicks (focused on lower limb activation).<br>2. Dry-land Exercises (15 mins):<br>• Rhythmic Coordination: Kneeling breaststroke leg kicks integrated with breathing practice.<br>• Skill Refinement: Deconstructed breaststroke leg kick practice, 15 reps × 3 sets.<br>• Motor Automation: Continuous breaststroke leg kick drills, 20 reps × 3 sets.<br>• Advanced Coordination: Continuous breaststroke leg kicks integrated with rhythmic breathing, 20 reps × 5 sets.<br>3. In-water Session (45 mins):<br>• Conditioning: Freestyle leg kicks: 6 laps.<br>• Equipment-Assisted Propulsion: Breaststroke leg kicks using a kickboard (focused on kick power and symmetrical extension; no breathing integration).<br>• Hydrostatic Balance: Static and dynamic floating practice (focused on maintaining horizontal body alignment).

4. Post-Session Free Play & Consultation (5 mins): Brief unstructured water time and communication with parents regarding technical progress.

5. Homework Assignment:

• Freestyle leg kicks: 300 reps total.

• Continuous breaststroke leg kicks: 30 reps × 5 sets.

Week 4, Session 2Theme: Simple Progression II (Breaststroke Leg Kicks)

1. Warm-up & Endurance (15 mins):

• General stretching and joint mobilization.

• 200 continuous freestyle leg kicks.

2. Dry-land Exercises (15 mins):

• Rhythmic Coordination: Kneeling breaststroke leg kicks with integrated breathing practice (5 mins).

• Motor Control: Continuous breaststroke leg kick drills: 20 reps × 3 sets.

• Advanced Coordination: Continuous breaststroke leg kicks with rhythmic breathing, 20 reps × 5 sets.

• Balance Training: Single-leg standing balance practice.

3. In-water Session (45 mins):

• Semi-water Drills (Poolside):

-Deconstructed breaststroke leg kicks (leaning on edge): 20 reps × 4 sets.

-Full breaststroke leg kick cycle with coordination (2 kicks per breath): 30 reps × 3 sets.

• In-water Drills (Holding Edge):

- Stationary breaststroke leg kicks with head above water: 30 reps × 3 sets.

- Stationary breaststroke leg kicks with rhythmic breathing (2 kicks per breath): 10 reps × 4 sets.

• Equipment-Assisted Propulsion: Breaststroke leg kicks using a kickboard (focused on linear progression; no breathing).

4. Post-Session Free Play & Consultation (5 mins): Brief unstructured water time and instructor-parent feedback.

5. Homework Assignment:

• Freestyle leg kicks: 300 reps total.

• Continuous breaststroke leg kicks: 30 reps × 5 sets.

Week 4, Session 3 Theme: Simple Progression II (Breaststroke Leg Kicks & Breathing Integration)

1. Warm-up & Endurance (5 mins):

• General stretching and joint mobilization.

• 200 continuous freestyle leg kicks.

2. Dry-land Exercises (30 mins):

• Intensive Lower Limb Training: Breaststroke leg kick drills combined with lower limb micro-muscle exercises.

• Coordination: Continuous breaststroke leg kicks integrated with breathing (2 mins).

• Motor Automation: Continuous breaststroke leg kick drills, 20 reps × 2 sets.

• Proprioception & Balance: Single-leg standing balance practice; Toe towel curls.

3. In-water Session (50 mins):

• Semi-water Coordination (Poolside): Breaststroke leg kick coordination with rhythmic breathing (2 kicks per breath), 20 reps × 3 sets.

• In-water Drills (Holding Edge):Stationary breaststroke leg kicks with rhythmic breathing (2 kicks per breath), 10 reps × 4 sets.

• Equipment-Assisted Progression:

-Breaststroke leg kicks using a kickboard (focused on propulsion; no breathing).

-Breaststroke leg kicks using a kickboard integrated with rhythmic breathing.

4. Post-Session Free Play & Consultation (5 mins): Unstructured water time and communication with parents regarding technical mastery.

5. Homework Assignment:

- Freestyle leg kicks: 300 reps total.
- Breaststroke leg kicks: 200 reps total.

Week 5, Session 1 Theme: Simple Progression III (Breaststroke Arm Strokes & Buoyancy Control)

1. Warm-up & Joint Mobilization: General stretching, joint flexibility exercises, and muscle activation.

2. Dry-land Exercises (15 mins):

- Lower Limb Consolidation: Full breaststroke leg kick cycles, 20 reps × 2 sets.
- Skill Acquisition (Upper Limb): Deconstructed breaststroke arm stroke mechanics (framework and recovery), 10 reps × 5 sets.
- Fine Motor & Balance: Toe towel curls; Single-leg standing balance practice.

3. In-water Session (40 mins):

- Warm-up Laps: Freestyle leg kicks, 4 laps.
- Equipment-Assisted Drills:

-Breaststroke leg kicks using a kickboard (no breathing).

-Breaststroke leg kicks with integrated buoyancy support: Using a kickboard and two back floats (back bubbles) while practicing rhythmic breathing.

4. Post-Session Free Play & Consultation (5 mins): Unstructured water time and communication with parents regarding the introduction of arm movements and buoyancy aids.

5. Homework Assignment:

- Freestyle leg kicks: 300 reps total.
- Breaststroke leg kicks: 300 reps total.

Week 5, Session 2 Theme: Simple Progression III (Breaststroke Arm Strokes & Motor Refinement)

1. Dry-land Exercises (40 mins):

- Fine Motor Training: Toe towel curls (intensive lower limb micro-muscle exercise), 50 reps × 3 sets.
- Lower Limb Consolidation: Full breaststroke leg kick cycles: 20 reps × 2 sets.
- Skill Acquisition (Upper Limb): Deconstructed breaststroke arm stroke mechanics, 10 reps × 4 sets.

2. In-water Session (40 mins):

- Semi-water Drills (Poolside): Continuous breaststroke leg kicks (leaning on the edge), 10 reps × 2 sets.
- Endurance Laps: Freestyle leg kicks: 4 laps.
- Equipment-Assisted Propulsion:

-Breaststroke leg kicks using a kickboard (no breathing).

-Breaststroke leg kicks using a kickboard integrated with rhythmic breathing.

3. Post-Session Free Play & Consultation (5 mins): Brief unstructured water time and instructor-parent feedback regarding coordination progress.

4. Homework Assignment:

- Freestyle leg kicks: 300 reps total.
- Breaststroke leg kicks: 300 reps total.
- Deconstructed breaststroke arm strokes: 100 reps total.

Week 5, Session 3 Theme: Simple Progression III (Introduction of In-water Arm Strokes)<br>1. Dry-land Exercises (35 mins):<br>• Skill Deconstruction: Deconstructed breaststroke arm stroke practice, 10 reps × 2 sets.<br>• Dynamic Integration: Full breaststroke arm stroke cycle practice, 10 reps × 3 sets.<br>• Objective: To internalize the trajectory of the "Out-sweep, In-sweep, and Recovery" phases.<br>2. In-water Session (45 mins):<br>• Semi-water Arm Drills (Poolside):<br>-Deconstructed breaststroke arm strokes (leaning on the edge).<br>-Full breaststroke arm stroke cycles.<br>• Semi-water Leg Drills: Full breaststroke leg kick cycles (to maintain motor memory).<br>• Equipment-Assisted Progression:<br>-Breaststroke leg kicks using a kickboard integrated with rhythmic breathing (Multiple sets).<br>3. Post-Session Free Play & Consultation (5 mins): Brief unstructured water time and instructor-parent feedback regarding the transition to upper-limb movements.<br>4. Homework Assignment:<br>• Breaststroke leg kicks: 300 reps total.<br>• Deconstructed breaststroke arm strokes: 100 reps total.
Week 6, Sessions 1, 2, & 3 Theme: Simple Progression III (Breaststroke Arm Strokes & Breathing Coordination)<br>1. Dry-land Exercises (15 mins):<br>• Upper Limb Coordination: Deconstructed breaststroke arm strokes integrated with rhythmic breathing: 10 reps × 3 sets.<br>• Fine Motor Training: Toe towel curls (lower limb micro-muscle exercise): 30 reps × 3 sets.2. In-water Session (45 mins):<br>• Semi-water Coordination (Poolside): Deconstructed breaststroke arm strokes integrated with rhythmic breathing (leaning on the edge).<br>• Equipment-Assisted Propulsion:<br>-Breaststroke leg kicks using a kickboard integrated with rhythmic breathing.<br>• Skill Consolidation (In-water): Independent stationary breaststroke arm stroke practice (focusing on water feel and the "Out-sweep/In-sweep" trajectory).<br>• Cool-down: Gentle freestyle leg kicks for muscle relaxation.<br>3. Post-Session Free Play & Consultation (5 mins): Brief unstructured water time and progress review with parents regarding the synchronization of arm movements and breathing.<br>4. Homework Assignment:<br>• Breaststroke leg kicks: 300 reps total.<br>• Deconstructed breaststroke arm strokes: 100 reps total.
Week 7, Sessions 1, 2, & 3 Theme: Simple Progression III (Breaststroke Arm Strokes & Advanced Breathing)<br>1. Dry-land Imitation & Warm-up (15 mins):<br>• Imitation Training: Mirroring deconstructed breaststroke arm stroke mechanics.<br>• Dynamic Drills: Full breaststroke arm stroke cycle practice: 10 reps × 3 sets.<br>• Fine Motor Skill: Toe towel curls for lower limb neuromuscular activation.<br>2. In-water Session (45 mins):

• Semi-water Refinement (Poolside): Full breaststroke arm stroke cycles (leaning on the edge), 10 reps × 5 sets.<br>• Equipment-Assisted Drills: Deconstructed breaststroke arm strokes using a kickboard (focused on the "catch" and "pull" phases).<br>• Halliwick-based Breathing: Basic rotation breathing exercises (focused on trunk stability and rhythmic exhalation).<br>3. Post-Session Free Play & Consultation (5 mins): Unstructured water time and communication with parents regarding the participant's imitation accuracy and breathing control.<br>4. Homework Assignment:<br>• Breaststroke leg kicks: 300 reps total.<br>• Deconstructed breaststroke arm strokes: 100 reps total.
Week 8, Sessions 1, 2, & 3 Theme: Basic Technique Learning I (Full Stroke Coordination)<br>1. Dry-land Imitation & Warm-up (15 mins):<br>• Fine Motor Skill: Toe towel curls (50 reps) for distal neuromuscular control.<br>• Upper Limb Refinement: Full breaststroke arm stroke cycles: 10 reps × 3 sets.<br>• Whole-body Integration: Dry-land practice of full breaststroke coordination (synchronizing arms and legs) integrated with rhythmic breathing.2. In-water Session (45 mins):<br>• Semi-water Consolidation (Poolside): Full breaststroke coordination drills (leaning on the edge), 10 reps × 5 sets to stabilize the movement sequence.<br>• Endurance Propulsion: Continuous breaststroke leg kicks using a kickboard (25m non-stop).<br>• Full Stroke Integration: Comprehensive breaststroke coordination practice (independent swimming for 20m).<br>• Cool-down: Gentle freestyle leg kicks for muscle relaxation and recovery.<br>3. Post-Session Free Play & Consultation (10 mins): Unstructured water play and strategic communication with parents regarding the transition to full-stroke swimming.<br>4. Homework Assignment:<br>• Full breaststroke coordination drills: 100 reps total.<br>• Full coordination integrated with breathing: 100 reps total.
Week 9, Sessions 1, 2, & 3 Theme: Basic Technique Learning II (Full Stroke and Breathing Integration)<br>1. Dry-land Imitation & Warm-up (15 mins):<br>• Fine Motor Skill: Toe towel curls (50 reps) for neuromuscular conditioning.<br>• Upper Limb Refinement: Full breaststroke arm stroke cycles: 10 reps × 3 sets.<br>• Whole-body Integration: Dry-land practice of full breaststroke coordination integrated with rhythmic breathing.<br>2. In-water Session (45 mins):<br>• Semi-water Consolidation (Poolside): Full breaststroke coordination drills with breathing (leaning on the edge), 10 reps × 5 sets.<br>• Endurance Propulsion: Continuous breaststroke leg kicks using a kickboard (25m non-stop).<br>• Technical Isolation: Independent breaststroke arm stroke practice (focusing on the "pull-and-breathe" timing).<br>• Full Stroke Mastery: Comprehensive breaststroke coordination practice (independent swimming for 20m).<br>• Cool-down: Gentle freestyle leg kicks for muscle relaxation.<br>3. Post-Session Free Play & Consultation (5 mins): Unstructured water time and instructor-parent feedback regarding stroke efficiency and respiratory rhythm.<br>4. Homework Assignment:

• Full breaststroke coordination with breathing: 100 reps total.<br>• Freestyle leg kick practice (for maintaining core stability).
Week 10, Sessions 1, 2, & 3Theme: Basic Technique Learning II (Full Stroke Consolidation & Respiratory Mastery)<br>1. Dry-land Imitation & Warm-up (15 mins):<br>• Neuromuscular Training: Toe towel curls (50 reps) for fine motor control.<br>• Upper Limb Refinement: Full breaststroke arm stroke cycles, 10 reps × 3 sets.<br>• Dry-land Coordination: Practice of full breaststroke coordination synchronized with rhythmic breathing to reinforce the motor program.<br>2. In-water Session (45 mins):<br>• Semi-water Coordination (Poolside): Full breaststroke coordination with integrated breathing (leaning on the edge): 10 reps × 5 sets.<br>• Endurance Propulsion: Continuous breaststroke leg kicks using a kickboard (25m non-stop) to enhance lower-limb stamina.<br>• Technical Isolation: Independent breaststroke arm stroke practice (focusing on the "pull-to-breathe" synchronization).<br>• Full Stroke Mastery: Comprehensive independent breaststroke swimming (20m bouts).<br>• Cool-down: Gentle freestyle leg kicks for metabolic recovery and muscle relaxation.<br>3. Post-Session Free Play & Consultation (5 mins): Brief unstructured water play and feedback session with parents focusing on stroke fluency and water confidence.<br>4. Homework Assignment:<br>• Full breaststroke coordination with breathing: 100 reps.<br>• Freestyle leg kicks: 300 reps (for core and hip flexor conditioning).
Week 11, Sessions 1, 2, & 3 Theme: Basic Technique Learning III (Introduction to Freestyle Arm Mechanics)<br>1. Dry-land Imitation & Warm-up (15 mins):<br>• Neuromuscular Training: Toe towel curls (50 reps).<br>• Skill Acquisition (Freestyle):<br>-Deconstructed freestyle arm stroke drills (entry, pull, and recovery): 30 reps × 3 sets.<br>-Full freestyle arm stroke cycle practice.<br>• Whole-body Integration: Dry-land practice of the full freestyle swimming motion to establish the cross-lateral motor pattern.<br>2. In-water Session (45 mins):<br>• Semi-water Refinement (Poolside): Deconstructed freestyle arm strokes (leaning on the edge), 30 reps × 5 sets.<br>• Equipment-Assisted Propulsion: Continuous freestyle arm strokes using a kickboard (25m non-stop) to practice unilateral and bilateral pulling.<br>• Lower Limb Conditioning: Continuous freestyle leg kick drills.<br>• Halliwick-based Rotation: Advanced longitudinal rotation exercises (focused on body roll and lateral stability).<br>3. Post-Session Free Play & Consultation (5 mins): Brief unstructured water time and instructor-parent feedback regarding the participant's adaptation to alternating arm movements.<br>4. Homework Assignment:<br>• Deconstructed freestyle arm strokes: 100 reps.<br>• Full freestyle arm stroke practice.

• Freestyle leg kick drills.
Week 12, Sessions 1, 2, & 3Theme: Basic Technique Learning IV (Full Freestyle Coordination & Mastery)<br>• Dry-land Imitation & Warm-up (15 mins):<br>• Neuromuscular Activation: Toe towel curls (50 reps).<br>• Advanced Coordination:<br>-Full freestyle arm stroke cycle drills: 30 reps × 3 sets.<br>-Full freestyle coordination integrated with rhythmic breathing: 30 reps × 5 sets.<br>• Objective: To solidify the kinetic link between alternating arm strokes and bilateral leg kicks.<br>2. In-water Session (45 mins):<br>• Semi-water Refinement (Poolside):<br>-Full freestyle coordination drills: 30 reps × 5 sets.<br>-Full coordination integrated with rhythmic breathing: 30 reps × 5 sets.<br>• Full Stroke Mastery: Continuous independent freestyle swimming (25m non-stop).<br>• Conditioning & Balance:<br>-Continuous freestyle leg kick drills.<br>-Advanced longitudinal rotation drills (focused on streamlined body position during breathing).<br>3. Post-Session Free Play & Consultation (5 mins): Final unstructured water play and summative feedback session with parents regarding the participant's overall progress and aquatic safety skills.<br>4. Homework Assignment:<br>• Full freestyle coordination practice: 100 reps.<br>• Full coordination integrated with breathing practice.<br>• Freestyle leg kick drills.

Table S2 Session Tracking Template

Participant Name: Recorded by: Location:<br>Date (YYYY/MM/DD): Session No.								
Session Components		Activity Details	Support Provided		Level of Engagement	Completion Rate	Skill Proficiency Rating	Remarks
			Teacher	Parent				
Dry-land Training		Warm-up Exercises						
		Stretching						
		Balance Training						
		Kicking Drills						
		Arm Movement Imitation						
Semi-water		Poolside Kicking Drills						
		Poolside Arm Drills						
Water Entry		Pre-entry Preparation						
		Entry Posture						
In-water Session	Phase I: Water Adaptation	In-water Instruction						
		Breathing Exercises						

	Phase II: Review & Consolidation	Review of Previous Session						
	Phase III: Skill Development	Kicking Drills						
		Arm Stroke Drills						
	Phase IV: Independent Propulsion	Independent Swimming						
Post-Session Activities		Independent play in shallow water						
		Promptly exiting water upon command						
Emotion								
Session Summary								
Session Focus								

This template was used by the instructors to monitor and record the fidelity of each of the 36 intervention sessions, tracking land-based, semi-water, and in-water components for each participant.

Table S3 Interview Guide for Guardians.

NO.	Questions
1	At what stage did you first notice that your child was different from other children?
2	What were the initial symptoms you observed?
3	At what age was your child officially diagnosed with ASD?
4	At what age did your child begin receiving intervention?
5	Which of your child's behaviors causes you the most concern?
6	Why did you agree to participate in this aquatic intervention experiment?
7	Which specific symptoms did you hope to improve through this experiment?
8	In which areas do you feel your child has improved through this experiment?
9	What progress was made at stages T1, T2, and T3, respectively? What surprised or pleased you the most?
10	Will you continue to let your child participate in swimming activities in the future?
11	Would you agree to participate in similar exercise intervention experiments in the future?
